# B Cell Repertoire Analysis Identifies New Antigenic Domains on Glycoprotein B of Human Cytomegalovirus which Are Target of Neutralizing Antibodies

**DOI:** 10.1371/journal.ppat.1002172

**Published:** 2011-08-11

**Authors:** Sonja Pötzsch, Nadja Spindler, Anna-Katharina Wiegers, Tanja Fisch, Pia Rücker, Heinrich Sticht, Nina Grieb, Tina Baroti, Florian Weisel, Thomas Stamminger, Luis Martin-Parras, Michael Mach, Thomas H. Winkler

**Affiliations:** 1 Nikolaus-Fiebiger-Zentrum für Molekulare Medizin Friedrich-Alexander Universität Erlangen-Nürnberg, Germany; 2 Institut für Klinische und Molekulare Virologie Friedrich-Alexander Universität Erlangen-Nürnberg, Germany; 3 Institut für Biochemie, Friedrich-Alexander Universität Erlangen-Nürnberg, Germany; 4 4-Antibody AG, Jena, Germany; Washington University School of Medicine, United States of America

## Abstract

Human cytomegalovirus (HCMV), a herpesvirus, is a ubiquitously distributed pathogen that causes severe disease in immunosuppressed patients and infected newborns. Efforts are underway to prepare effective subunit vaccines and therapies including antiviral antibodies. However, current vaccine efforts are hampered by the lack of information on protective immune responses against HCMV. Characterizing the B-cell response in healthy infected individuals could aid in the design of optimal vaccines and therapeutic antibodies. To address this problem, we determined, for the first time, the B-cell repertoire against glycoprotein B (gB) of HCMV in different healthy HCMV seropositive individuals in an unbiased fashion. HCMV gB represents a dominant viral antigenic determinant for induction of neutralizing antibodies during infection and is also a component in several experimental HCMV vaccines currently being tested in humans. Our findings have revealed that the vast majority (>90%) of gB-specific antibodies secreted from B-cell clones do not have virus neutralizing activity. Most neutralizing antibodies were found to bind to epitopes not located within the previously characterized antigenic domains (AD) of gB. To map the target structures of these neutralizing antibodies, we generated a 3D model of HCMV gB and used it to identify surface exposed protein domains. Two protein domains were found to be targeted by the majority of neutralizing antibodies. Domain I, located between amino acids (aa) 133–343 of gB and domain II, a discontinuous domain, built from residues 121–132 and 344–438. Analysis of a larger panel of human sera from HCMV seropositive individuals revealed positivity rates of >50% against domain I and >90% against domain II, respectively. In accordance with previous nomenclature the domains were designated AD-4 (Dom II) and AD-5 (Dom I), respectively. Collectively, these data will contribute to optimal vaccine design and development of antibodies effective in passive immunization.

## Author Summary

The development of antibodies is a major defense mechanism against viruses. Understanding the repertoire of antiviral antibodies induced during infection is a necessary prerequisite to defining the protective activities of an antiviral antibody response. The isolation of antigen specific memory B cells and subsequent stimulation to antibody producing cells provides a powerful tool to study the antibody repertoire in infected individuals. We have used this approach to analyze the antibody repertoire against glycoprotein B (gB) of human cytomegalovirus (HCMV), a major antigen for the induction of antiviral antibodies during infection and a constituent of experimental vaccines in humans. We find in different infected individuals that the vast majority of gB-specific B cells produce antibodies that cannot neutralize free virus. Antibodies with antiviral capacity target two domains of gB that have not been previously identified. The identification of these new antigenic domains was possible with the aid of a 3D molecular model of HCMV gB. Our results will be useful for vaccine development since comparison of the immune response after natural infection with that induced by vaccination can be readily accomplished. Moreover, neutralizing human monoclonal antibodies could constitute powerful therapeutics to combat the infection in populations at risk for HCMV disease.

## Introduction

Human cytomegalovirus (HCMV) is an important, ubiquitously occurring, human pathogen in immunocompromised hosts. The virus can cause severe disease in transplant recipients [1]. In large parts of the world HCMV is also the most common viral infection acquired *in utero*. In the USA and Europe an estimated 0.2%–1.2% of all live born infants are infected with HCMV [2], [3]. Congenital HCMV infection is a leading cause of sensorineural hearing loss in children and the leading infectious cause of central nervous system damage in children [4], [5]. As a consequence of the importance of congenital HCMV infection for public health, the Institute of Medicine of the National Academy of Sciences, USA, has ranked the development of a HCMV vaccine as a top priority [6].

As with all successful antiviral vaccines, induction of an efficient antibody response will be crucial for the success of such a vaccine [7]. Importantly, an effective anti-HCMV vaccine will need to protect the vaccine from HCMV infection/disease as well as in the case of pregnant women, the developing fetus. Transfer of protective maternal antibodies to the fetus will be critical in this respect and a study of passive transfer of immunoglobulins to pregnant mothers has supported a role of antibodies in reducing the risk for congenital infection [8], [9]. Also, naturally acquired maternal immunity contributes to prevention of congenital HCMV infection [10]. Although correlates of protection from HCMV infection are poorly understood, it can be predicted that humoral immune responses to the envelope glycoproteins will be particularly important since antibodies directed against these antigens can neutralize virus infectivity directly and/or induce immunoglobulin Fc-receptor mediated effector functions such as antibody dependent cytotoxicity and/or complement mediated effects which can lead to elimination of infected cells [11].

HCMV is a highly complex virus harboring more than 20 different glycoproteins in its envelope [12], [13]. With respect to induction of neutralizing antibodies during natural infection, the glycoprotein (g) B dominates, but additional antigens such as the gM/gN complex and the gH/gL complex have also been identified as highly immunogenic [14]–[16]. Antibodies directed against gB can be detected in all naturally infected individuals [17]. Moreover, a major fraction of neutralizing antibodies in human sera seems to be directed against gB and the overall neutralizing capacity in sera from HCMV-seropositive donors correlates with anti-gB antibody titer [18]. In addition, anti-gB antibodies are effective in preventing cell-to-cell spread [19]. In the guinea pig CMV model, immunization with gB DNA vaccines confers protection from infection [20]. In a recent study protection from brain pathology in murine cytomegalovirus (MCMV) infected mice was accomplished by passive transfer of a gB-specific monoclonal antibody (mab) [21]. Thus, gB is an attractive antigen for inclusion in a human vaccine and has been part of a number of experimental vaccines [22], [23]. In fact, a recent phase 2 trial using recombinant gB as vaccine has shown significant protection from infection [24]. However, the spectrum of anti-gB antibodies developing during infection remains poorly defined.

HCMV gB is an essential viral protein which is involved in the early events of infection. It has been shown to bind to a variety of cell surface molecules such as heparan sulfate proteoglycans, integrin heterodimers and platelet-derived growth factor-α receptor [25]–[27]. In addition, HCMV gB has been shown to mediate fusion of viral and cellular membranes [28]–[30]. The protein is essential for viral entry and cell-to-cell spread but not for virion attachment, assembly or egress [31]. Three antigenic domains (AD) have been described previously. AD-1 consists of approximately 80aa between positions 560 and 640 of gB of HCMV strain AD169 [32]. It is the immunodominant region of gB since nearly all sera from HCMV-infected individuals recognize AD-1 [33]. Antibodies that bind to AD-1 can have virus neutralizing capacity as indicated by the fact that a number of AD-1-specific human mabs have been isolated which show various degrees of neutralizing activity [34], [35]. Polyclonal AD-1-specific antibodies, purified from human serum, are incapable of completely neutralizing HCMV even at high concentration, indicating that AD-1 is bound by antibodies with widely differing neutralizing activity [36]. It has been suggested that the competitive binding of neutralizing and non-neutralizing antibodies to AD-1 may represent a mechanism to evade efficient neutralization of cell free virus [36].

AD-2, located at the extreme amino terminus of the protein, consists of at least two distinct sites between aa 50 and 77 of gB. Site I is common to all HCMV strains and induces neutralizing antibodies, whereas the aa sequence of site II differs between strains and is recognized by strain specific antibodies which are incapable of neutralization *in vitro* [37]. The overall immunogenicity of AD-2 is lower than that of AD-1 since only about 50% of human sera from HCMV-infected donors have antibodies against this determinant [17]. An additional linear aa sequence, AD-3, recognized by gB-specific antibodies in human sera includes epitopes at the intraluminal/intraviral part of the molecule [38], [39]. However, antibodies binding to these determinants are non-neutralizing as can be expected from the localization of AD-3 within the molecule. Additional protein domains which are bound by murine mabs have been identified, but whether these regions are relevant in the context of antibody response during natural infection is unknown [40]. The gB-specific human monoclonal antibodies for which the binding sites has been identified react with either AD-1 or AD-2 [35], [41].

Overall, there are significant gaps in our knowledge of antibody epitopes on gB. Given the size of the gB protein it seems highly likely that additional antigenic domains exist on gB. Defining these sites will not only provide an antigenic map of this important protein, it will also be helpful for monitoring the response to vaccination for production of antibodies with binding profiles similar to natural infection.

We comprehensively analyzed the human antibody repertoire against gB as it is developed during infection. To this end we isolated gB-specific memory B cells from different healthy HCMV-seropositive donors and activated the cells at the clonal level to immunoglobulin production. The produced antibodies were tested for reactivity with gB and parts thereof and in *in vitro* neutralization assays. Our results revealed that most of the anti-gB antibodies produced during infection failed to neutralize cell-free virus. In addition, and perhaps more importantly, we find that the vast majority of anti-gB antibodies with potent neutralizing capacity recognize two protein domains which have not been identified previously as target sites.

## Results

### The antibody repertoire against gB is dominated by antibodies that do not neutralize virus and those which bind to unknown protein domains

We intended to comprehensively analyze the human IgG anti-gB memory B-lymphocyte repertoire established by healthy HCMV infected individuals in terms of epitope specificity as well as neutralizing capacity. To this end we used the complete extraviral part of gB, as it is used in vaccination trials [24], for sorting of IgG positive memory B-lymphocytes (CD19^+^/CD27^+^) binding to fluorochrome-labeled gB by flow cytometry (Fig. S1A). In a first set of experiments we analyzed the possibility to identify gB-specific memory B cells in 15 seropositive individuals. Frequencies of gB-binding, IgG-positive memory B cells among all IgG-positive memory B cells ranged from 0.33 to 1.4%, being in the range of frequencies of IgG memory B-lymphocytes against other viral antigens [42] (Fig. S1B). Among HCMV seronegative individuals, gB-binding memory B cells were detectable but with considerably lower frequency. These cells might bind gB unspecifically or may represent part of the natural antibody repertoire [43]. Sorted gB-binding B cells were activated and immortalized at the clonal level by an *in vitro* culture system using CpG oligonucleotides and EBV [44]. Among clonal cultures with IgG secretion 40–95% (mean 63%) of cultures showed IgG binding to gB in ELISA, substantiating a high degree of specificity in the cell sorting process (data not shown).

Seven donors were selected for further analysis. The anti-gB antibody titer was comparable in this group (Fig. S2A) while the neutralization titer varied significantly, which is not uncommon for HCMV-infected individuals (Fig. S2B). From these 7 donors we were able to analyze a total of 888 clonal IgG gB-binding culture supernatants for epitope specificity towards the well-known antigenic domains AD-1 and AD-2 [45] as well as neutralizing capacity against HCMV AD169 on fibroblasts. With respect to binding of gB protein domains we found a high frequency of antibodies binding to the AD-1 epitope (mean 38.1%) in all individuals correlating with earlier findings that up to 50% of gB-specific IgG in sera might be directed against the AD-1 epitope (Table 1) [38]. Interestingly, only few of the AD-1-specific antibodies were able to neutralize HCMV *in vitro* (mean 2.0%, range 0–6%; Table 1). The frequency of clones producing AD-2-specific IgG was low (Table 1) and in only 3 out of 7 individuals were we able to retrieve AD-2-specific memory B cells, correlating with earlier data that this specificity is found only in approximately 50% of HCMV infected individuals [17]. None of the rare AD-2-specific antibodies was neutralizing *in vitro*. A high frequency of clones from all individuals did not react with either AD-1 or AD-2 (range 40–86%; Table 1). Importantly, among these antibodies a significant number was able to neutralize HCMV *in vitro* (17% out of 429 clones; Table 1). A summary of the neutralizing capacity of the gB-specific B-cell supernatants of 5 donors from which we were able to isolate B cells that secreted neutralizing antibodies is shown in Fig. S2C. We conclude from this analysis that the memory B-cell repertoire against gB is dominated by antibodies that do not neutralize the virus and that most neutralizing antibodies bind to a so far unknown antigenic site.

**Table 1:** Epitope specificity and neutralizing activity of IgG HCMV-gB specific antibodies.

			epitope specificity									
			AD-1		AD-2		Dom II/AD-4		Dom I/AD-5		unknown	
Donor	number^a^	nt b (%)	total^b^ (%)	nt^c^ (%)	total b (%)	nt^c^ (%)	total^b^ (%)	nt^c^ (%)	total^b^ (%)	nt^c^ (%)	total^b^ (%)	nt^c^ (%)
AB	78	3	43	0	0	0	0	0	3	100	54	0
SA	34	0	26	0	0	0	0	0	0	0	74	0
JN	72	1	42	0	1	0	0	0	1	100	56	0
TJ	92	0	58	0	2	0	0	0	0	0	40	0
NT	250	11	14	5	0	0	25	87^d^	18	100 e	43	0
TW	124	5	56	6	0	0	2	100	0	0	42	0
TS	238	2	28	3	1	0	14	100^f^	3	100^g^	54	0
mean, (range)	127 (34–250)	3.1 (0–11)	38.1 (14–56)	2.0 (0–6)	0.6 (0–2)	0	5.9 (0–25)	95.7 (87–100)		100	51.9 (40–74)	0

a total number of gB specific clones analyzed.

b % of all clones.

c percentage of epitope specific clones with 50% neutralization at concentrations of 3 µg or less. Every antibody was tested at least twice with similar results.

d 13 of 15 clones that could be tested.

e 12 of 45 clones that could be tested.

f 1 of 34 clones that could be tested.

g 1 of 7 clones that could be tested.

### Neutralizing human monoclonal antibodies bind to a region between residues 100–448 of gB

**Figure 1:**
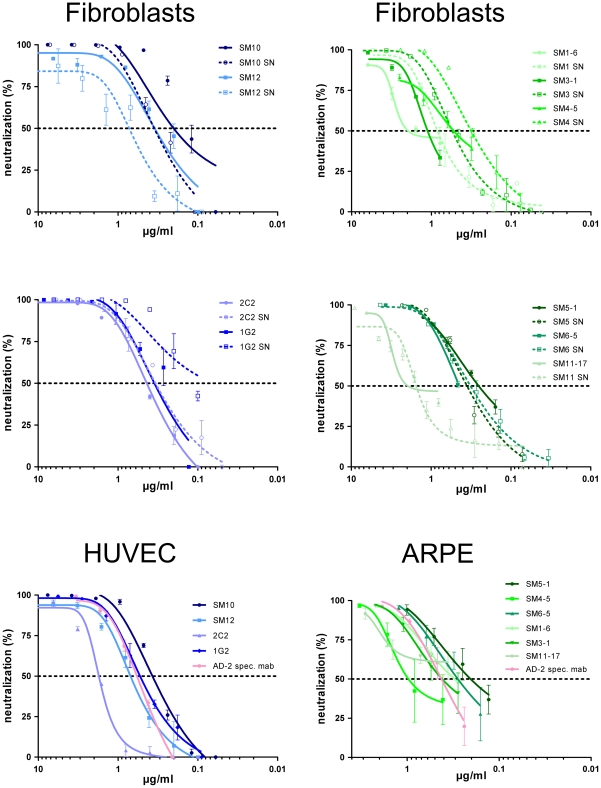
Neutralization capacity of Dom I- and Dom II-specific mabs. The indicated B-cell supernatants (SN) and recombinant IgGs were tested for neutralization using different target cells. The AD-169 derived recombinant virus was used for fibroblasts as target cells. Human umbilical cord endothelial cells (HUVEC) and human retinal pigment epithelial cells (ARPE) were infected with the HCMV strain TB40E [76]. Color code of the antibodies according to their target structure on gB as shown in Fig. 3. Every antibody was tested at least 3 times and representative results are shown.

Next, we attempted to map the target sites of antibodies that did not react with the known antigenic sites on gB. To obtain a consistent supply of mabs, the Ig-genes from 10 selected B-cell clones that secreted neutralizing antibodies unreactive with AD-1 and AD-2 were cloned and expressed in recombinant systems as IgG1 molecules (Table 2). We choose the IgG1 subtype since 8 of the 10 selected B-cell clones secreted IgG1, whereas 2 secreted IgG3. The recombinant antibodies showed comparable neutralizing activity to the B-cell supernatants when tested on fibroblasts as target cells indicating that the Fc-part of the IgG is not significantly contributing to the *in vitro* neutralizing activity of the antibodies (Fig. 1). 50% neutralization was achieved at concentrations of 0.2–1.3 µg/ml (Table 2). Importantly, the recombinant antibodies were also able to neutralize virus on endothelial, epithelial and dendritic cells with comparable activities. Representative results for Group A mabs (see next paragraph) on endothelial cells and Group B mabs on epithelial cells are shown in Fig. 1 and the data for all mabs are summarized in Table 2.

**Table 2:** Neutralization capacity and reactivity pattern of recombinantly expressed human monoclonal antibodies.

		50% neutralization activity (µg/ml) a					
Group and Clone	original IgG isotype	fibroblasts	endoth. cell	epithel. cell	dendritic cell	reactivity with domain I peptide^b^	reactivity with domain II peptide^b^
SM10	IgG3/κ	0.1	0,35	1,0	n.d.	+	-
1G2	IgG3/λ	0.2	0,5	1,5	n.d.	+	-
SM12	IgG1/κ	0.4	0,67	n.d.	n.d.	+	-
2C2	IgG1/κ	0.6	1,8	n.d.	n.d.	+	-
SM1-6	IgG1/λ	1.3	1.3	0.3	1.3	-	+
SM3-1	IgG1/λ	1.2	1.0	0.4	1.0	-	+
SM4-5	IgG1/λ	0.6	0.8	0.9	0.4	-	+
SM5-1	IgG1/λ	0.3	0.2	0.2	0.3	-	+
SM6-5	IgG1/λ	0.5	0.4	0.3	0.5	-	+
SM11-17	IgG1/λ	1.0	1.0	0.3	1.3	-	+

a every antibody was tested at least 3 times with similar results.

b tested by immunofluorescence on transiently transfected Cos7 cells.

**Figure 2:**
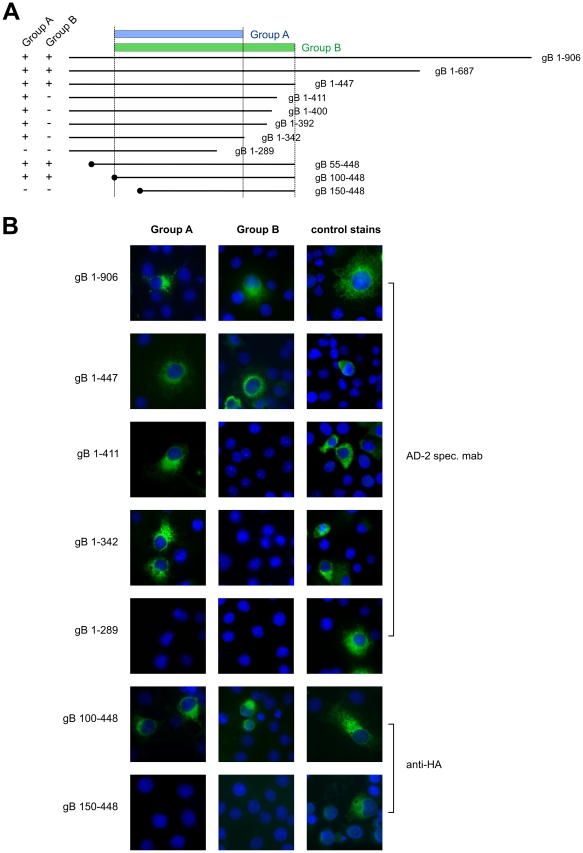
Antibody recognition of gB deletion mutants. (A) The parts of gB expressed by deletion mutants are represented by lines and the encompassed residues are given. Peptides starting with amino acid 1 of gB include the authentic signal sequence. Peptides starting at internal parts of gB contain a heterologous signal sequence and a HA-epitope tag at the amino terminus, indicated by the black circle. Reactivity of the respective gB peptides with different groups of monoclonal antibodies is indicated on the left of the figure and the minimal protein domains recognized by Group A and Group B mabs is indicated at the top. (B) Cos7 cells were transfected with the indicated plasmids and 48 h later processed for immunofluorescence using culture supernatants from Group A or Group B specific B-cell clones. Control stains for protein expression included a mab directed at gB-AD2 (aa 68-77), and an anti-HA mab as indicated at the right.

The fact that all tested antibodies were not reactive in western blot analyses using extracellular HCMV particles as antigen indicated that the intact three-dimensional protein conformation was crucial for binding (data not shown). Therefore, we used indirect immunofluorescence of transiently expressed fragments of gB in Cos7 cells to obtain further information on the binding sites of the mabs. Two distinct reactivity patterns were observed for the mabs (Group A and Group B in Fig. 2). Full length gB 1-906 and fragments as short as gB 1-447 were reactive with the entire set of mabs (Fig. 2). For Group A mabs gB residues 100–342 were sufficient for binding whereas for Group B antibodies a larger fragment of gB comprising residues 100–448 was required for binding, indicating that the gB region between aa 100–448 harbors at least two distinguishable antibody target sites.

### The generation of a structure model of gB reveals two globular protein domains within the antibody binding region of gB

**Figure 3:**
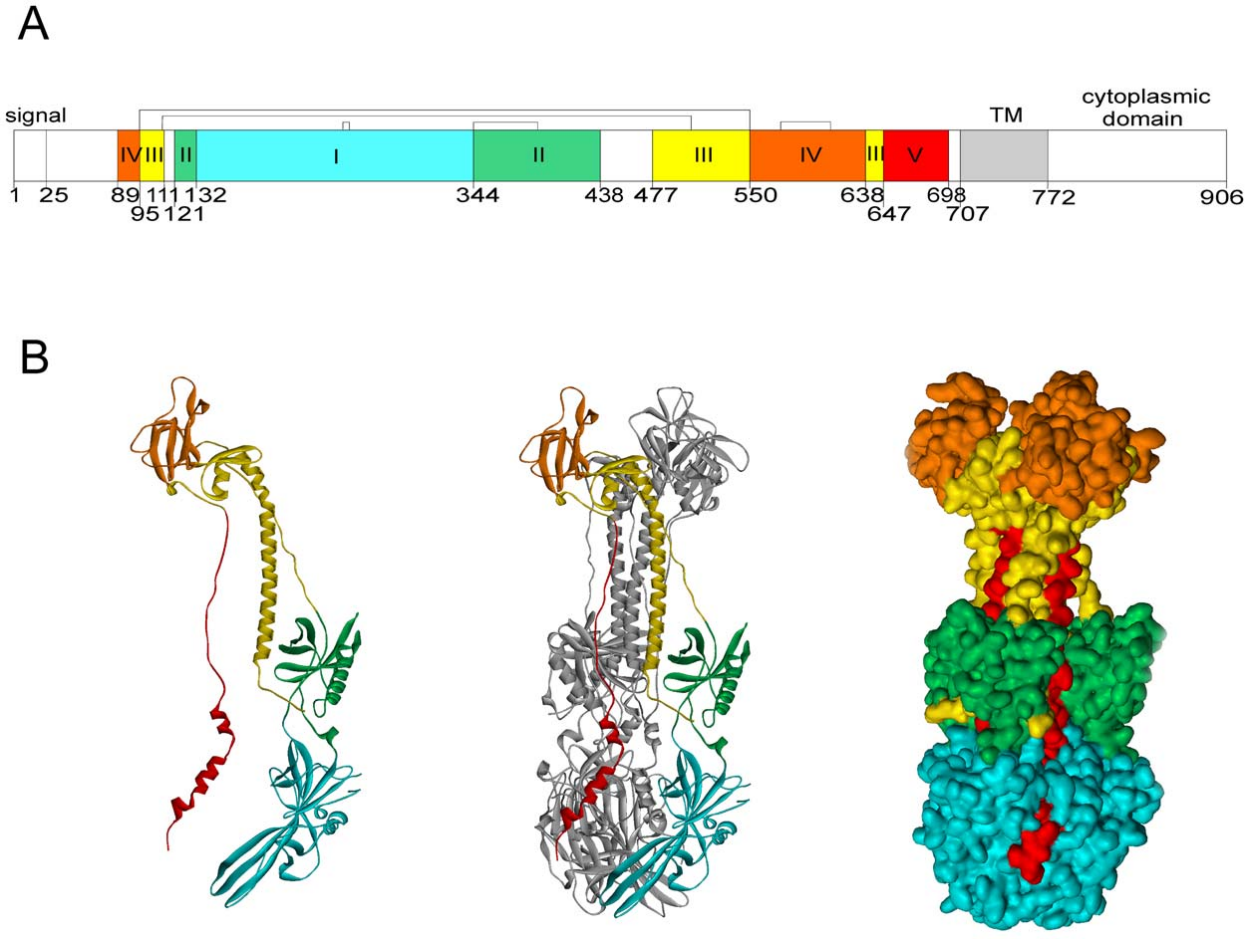
Domain architecture of HCMV gB. (A) The regions representing individual domains are displayed in different colors in analogy to the HSV gB structure by Heldwein et al. [46] and the numbers of the starting residues are given. Brackets indicate disulfide bonds. Signal: signal sequence, TM: transmembrane helix. (B) Ribbon diagram of a gB monomer (left), trimer with two protomers shown in grey (middle) and accessible surface representation of the trimeric gB (right). Coloring scheme according to (A).

In order to obtain further information on potential structural domains of gB in the region between aa 100–448, we generated a three dimensional model of the trimeric conformation of the ectodomain of HCMV gB strain AD169 based on the crystal structure of HSV-1 gB [46] (Fig. 3). According to the model, both the overall structure of the HCMV gB monomer and the organization of the subunits in the trimer were highly similar to that of HSV-1 gB, as expected from the degree of sequence similarity of gB between human herpesviruses (28% identity and 40% similarity between HSV-1 gB and HCMV strain AD169 gB). The individual domains I to V, which were previously defined based on the HSV-1 gB structure, can be clearly identified from the homology model of HCMV gB. With respect to potential antibody binding structures, the protein domains (Dom) I and II were of particular interest since they are located within aa 100–448 of gB. Dom I (Ile_133_ to Thr_343_) constitutes part of the trimer interface and is located proximal to the membrane, potentially containing the fusion domain [47]. The discontinuous Dom II is composed of two separate segments comprising residues Leu_121_ to Asn_132_ and Cys_344_ to Ser_438_ (Fig. 3). Either domain contains a single disulfide bond which helps to stabilize the conformation of the respective protein domain [48].

**Figure 4:**
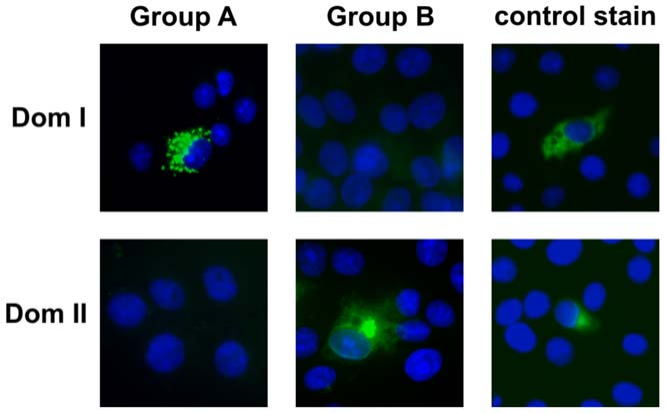
Identification of gB Dom I and Dom II as antibody binding structures. Plasmids expressing Dom I or Dom II were transfected into Cos7 cells and protein expression was analyzed 48 h later by indirect immunofluorescence using Group A or Group B mabs and a control stain (anti-HA antibody).

To investigate Dom I and Dom II for antibody binding, expression plasmids were constructed which allowed for the synthesis of either domain in eukaryotic cells. In both cases the cloning strategy involved the attachment of a HA epitope tag at the amino terminus of the respective peptide in order to facilitate detection. Dom I comprised aa 133–343 of gB. The Dom II-specific peptide consisted of the gB-specific residues 112–132 and 344–438 joined by a 5 aa (Ile-Ala-Gly-Ser-Gly) synthetic linker sequence. Following transient expression of the peptides in Cos7 cells, antibody binding was analyzed by indirect immunofluorescence. All four antibodies from Group A were reactive with the Dom I-specific peptide, whereas all Group B mabs recognized Dom II (Fig. 4 and Table 2). The crystal structure of HSV-1 gB as well as the model of HCMV gB predict that for Dom I and Dom II discontinuous sequence stretches are essential for formation of either domain. Shorter protein fragments would be expected not to fold correctly. We tested this assumption by expressing a Dom II variant with five amino acids deleted at the carboxyl-terminus and observed complete loss of binding of all Group A-specific antibodies. Likewise, the continuous part of Dom I (residues 140–255) showed no antibody binding capacity (data not shown). Thus, further resolution of antibody epitopes will be possible only by generation of point mutants in Dom I and Dom II, respectively. Having identified Dom I and Dom II as new targets for neutralizing antibodies, we re-tested clonal antibody supernatants from 4 individuals to obtain information about the overall frequency of Dom I- and Dom II-specific antibodies in HCMV infected individuals. As shown in Table 1, the frequency of Dom I and Dom II specific memory B cells was variable among different donors and in most cases considerably lower as compared to AD-1-specific B cells.

In summary, the repertoire analysis revealed that most anti-gB IgG antibodies derived from memory B cells are non-neutralizing. Among those antibodies that neutralized HCMV *in vitro,* most antibodies reacted against two previously uncharacterized regions of the gB protein. Addition of complement had no influence on the neutralizing capacity of the recombinant antibodies. Moreover, when a selected set (n = 10) of non-neutralizing antibodies directed against different antigenic domains of gB was tested in concentrations up to 5 µg/ml in the presence of complement we observed no significant increase in neutralization capacity which is in agreement with previous reports on gB-specific human mabs (data not shown) [35]. However, we cannot completely rule out a moderate enhancing effect of complement for some antibodies, especially those of the IgG3 subtype.

### Dom I and Dom II mabs function during a postadorption step of the infection

**Figure 5:**
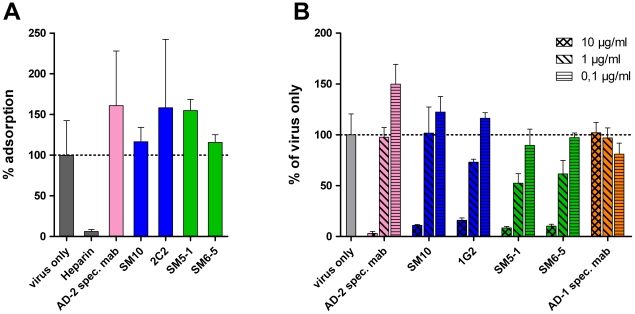
Mechanistic aspects of virus neutralization by Dom I and Dom II antibodies. (A) Virus (m.o.i. 0.5) was incubated with the indicated antibodies (10 µg/ml for Dom I-, and Dom II-specific mabs, 5 µg/ml for the AD-2-specific mab C23, 2 µg/ml heparin) for 1 h at 37°C and cooled to 4°C. The virus/antibody mixture was added to HFF and incubated for 1 h at 4°C. Lysates were prepared and processed for quantitative real time PCR analysis. The virus only sample was set to 100% and used to calculate the remaining samples. (B) HFF were adsorbed with virus at a m.o.i. of 0.2 at 4°C for 1 h. Antibody at the indicated concentrations was added and the culture was shifted to 37°C. Extent of infection was analyzed 48 h later and calculated relative to the virus only control (100%). Color code of the antibodies according to their target structure on gB as shown in Fig. 3.

gB has been postulated to be involved in receptor binding of HCMV and entry [26], [49], [50]. We therefore analyzed at which stage of the infection the mabs exerted their action. To assay influence on attachment, virus/antibody mixtures were added to target cells at 4°C and the number of HCMV DNA copies attached to the cells was determined by quantitative real time PCR. Neither Dom I- nor Dom II-specific antibodies inhibited attachment of virions, indicating that the mabs did not block receptor binding of HCMV (Fig. 5A). In accordance with previous reports, heparin almost completely prevented virus attachment, whereas the AD-2-specific antibody C23 had no effect on virus binding [27], [51]. The slight increase of bound virus in the presence of some antibodies as compared to control might reflect deposition of antibody/virus aggregates on the surface of cells. We also determined activity of the mabs towards virus that is already adsorbed to cells. To this end, virus was preadsorbed to cells for 1 h at 4°C before antibody was added. Both Dom I- and Dom II-specific antibodies were capable of neutralizing HCMV at a postadsorption step, whereas non-neutralizing antibodies had no effect (Fig. 5B). The higher antibody concentration that was required to completely neutralize adsorbed virus is explained by the need to block fusion of an already adsorbed virus particle.

### Neutralization of Dom I and Dom II antibodies is not influenced by non-neutralizing antibodies

**Figure 6:**
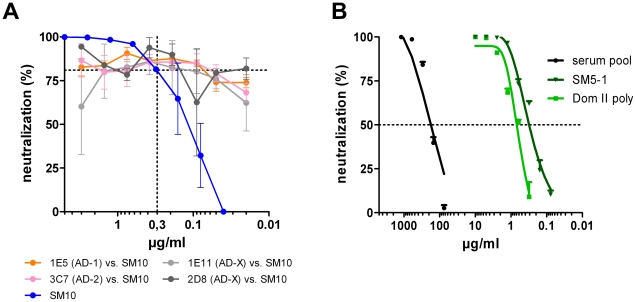
Influence of non-neutralizing antibodies on neutralizing capacity of antibodies. (A) SM10 at a concentration of 0.3 µg/ml was mixed with the indicated non-neutralizing antibodies (AD-X  =  unknown binding site on gB) at various concentrations and incubated with virus for 1 h. The virus/antibody mixture was added to HFF and neutralization was assayed 48 h later. (B) Neutralization capacity of the serum pool, the affinity purified Dom II-specific antibodies (Dom II poly) and a Dom II-specific mab (SM5-1). Shown are representative results from at least 3 independent analyses.

Competitive binding of neutralizing and non-neutralizing human mabs has been described for gB [35]. We tested whether a similar phenomenon occurs for Dom I- or Dom II-specific neutralizing antibodies. A total of 14 non-neutralizing mabs, directed against different antigenic regions of gB was analyzed in neutralization assays for competition with Dom I- or Dom II-specific mabs (SM10, 1G2, SM5-1). In no case did we observe a reduction in neutralizing capacity by addition of non-neutralizing mabs. The result of a representative analysis is shown in Fig. 6A. Although these data indicated that reduction of neutralizing activity by competing non-neutralizing mabs is not common for Dom I- and Dom II-specific antibodies, it is difficult to assess the relevance of this finding for the situation in human sera. For AD-1-specific antibodies it has clearly been shown that the sum of antibodies, as present in human sera, is not capable of completely neutralizing infectious virus, indicating that non-neutralizing antibodies can constitute a significant fraction of the entire AD-1-specific IgG fraction [36]. To obtain more information on the neutralizing capacity of Dom II-specific antibodies in human sera, we purified Dom II as a GST fusion protein following expression in *E. coli* (Fig. S3A). The bacterially derived peptide retained the capacity to bind all Group B mabs and the affinity of mab SM5-1 was similar for gB and Dom II (Fig. S3B). Circular dichroism spectra indicated a homogeneous three-dimensional structure (Fig. S3C). Using this protein, polyclonal Dom II-specific antibodies were affinity-purified from pooled human sera (Fig. S4). The affinity-purified IgG preparation was then tested in neutralization assays. 50% neutralization of input viral infectivity was achieved with an IgG concentration of approximately 0.2 µg/ml which is within the same concentration range as the Dom II-specific mabs (Fig. 6B). In summary, these data provided evidence that gB Dom II not only represents an immunogenic domain on gB, but also that antibodies binding to it in general have potent neutralizing capacity. Similar experiments using Dom I could not be carried out since the Dom I domain does not fold correctly after prokaryotic expression and thus antigen for affinity purification of antibodies could not be generated.

### Dom I and Dom II show different positivity rates in sera from infected donors

**Figure 7:**
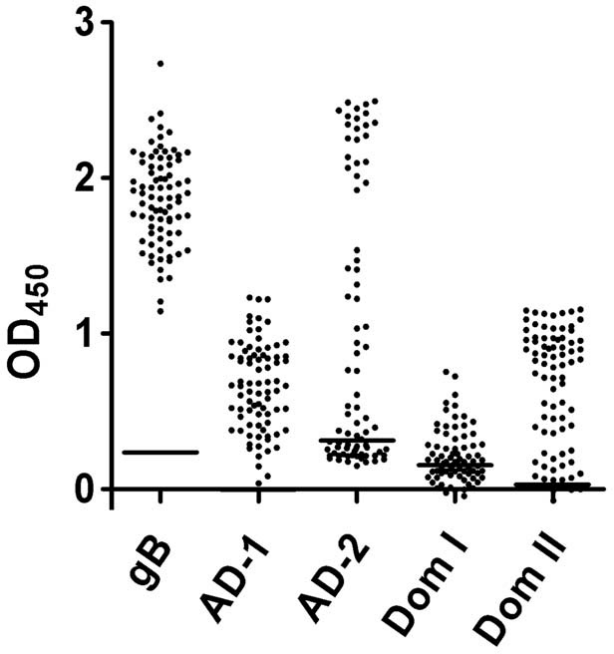
Recognition of the antigenic domains of gB by human sera. 80 randomly selected sera from HCMV seropositive individuals were analyzed in an ELISA for reactivity against recombinant gB (gB) and the antigenic domains 1 (AD-1), 2 (AD-2) and the structural domain I and II (Dom I, Dom II), respectively. The horizontal line represents the cutoff for the individual antigens. The cutoff for each antigen was defined as mean +2 SD of 10 HCMV-negative sera.

Previous analyses of human sera for recognition of gB domains have revealed differential rates of antibody reactivity for the individual antigenic domains. Whereas almost 100% of infected individuals develop antibodies against AD-1, only 50% show reactivity against AD-2 [17]. To obtain information on the frequency of recognition of Dom I and Dom II we determined antibody reactivity against these domains in a larger serum panel of HCMV infected individuals and compared it to the known antigenic domains. A total of 80 randomly selected sera from HCMV seropositive individuals, as determined by a commercially available ELISA test, was analyzed. Ten sera from HCMV negative donors served as controls. Within the serum panel from HCMV-seropositive individuals, reactivity for gB was 100%, underscoring the antigenicity of this protein (Fig. 7). In accordance with our previous observations, positive reaction with AD-1 was also 100% and 57% of the sera contained antibodies against AD-2 [17]. Dom I was recognized by 55% whereas 94% of the specimens reacted with Dom II. Thus, Dom I and Dom II represent antigenic domains on gB which induce antibodies with high frequency during infection. For the sake of consistency in nomenclature of gB antigenic domains, they were designated AD-4 (Dom II) and AD-5 (Dom I).

## Discussion

We have used recombinant gB to analyze the antibody repertoire derived from activated memory B cells of healthy HCMV seropositive individuals. The donors were not selected for hight titers of anti-gB or neutralizing antibodies against HCMV in order to obtain an unbiased result. Our results reveal a number of new findings with respect to the anti-gB response in humans.

The vast majority of anti-gB antibodies did not show antiviral activity in *in vitro* assays. Among the seven donors that were tested comprehensively, the percentage of neutralizing antibodies among the gB binders ranged from 0% to 11% (mean 3%). Previous studies using adsorption of antibodies to gB have noted that in some individuals the overall neutralization capacity cannot be reduced, indicating that in these cases non-gB specific antibodies are major components of the neutralizing antibody response [18]. Repertoire analysis in other viral systems have also noticed an excess of binding versus neutralizing antibodies [44], [52], [53].

At this time we can only speculate on a potential role of the non-neutralizing antibodies on the infection *in vivo*. Besides being irrelevant for the infection, non-neutralizing antibodies might have positive or negative effects. Effector functions mediated via the Fc-portion of the antibodies such as ADCC and/or complement fixation may contribute to elimination of infected cells and thus could lead to enhanced clearance of these cells. That such mechanisms are operative *in vivo* has been shown in different viral systems, including herpesviruses, but not for HCMV [54], [55]. On the other hand, infection-enhancing effects could be contributed by mechanisms such as competition of non-neutralizing antibodies for binding to neutralizing epitopes or enhanced infection of Fc-receptor bearing cells such as monocytes, which is one of the major target cell population for HCMV infection *in vivo* [56].

Whereas all anti-gB mabs recognized mammalian cell expressed gB aa 1-687, only 40% to 50% of anti-gB antibodies were reactive with a shorter fragment expressing aa 1–447 indicating the presence of epitopes in gB between aa 448 and 687. These antibodies were negative for recognition of the bacterial fusion protein containing AD-1 (aa 484–650). Thus, the location of the antibody binding site(s) remains unknown. There is the possibility that residues 447–484 and 650–687 contain additional epitopes since they are not represented by the AD-1 fusion peptide. However, this seems unlikely. The more plausible explanation for our finding is that the region between aa 447–687 contains additional conformational epitopes that are not formed in the bacterial fusion protein. Our previous analyses have shown that AD-1 induces a multitude of antibodies with different binding characteristics, even when assayed as bacterial fusion proteins or synthetic peptides [33]. Thus, it would be not surprising that this region of gB contains additional epitopes which are present only on the native protein. The large fraction of antibodies falling in this category also supports this possibility. A corresponding protein region of HSV-1 gB was found to contain a “pseudocontinuous” epitope, further supporting our interpretation [57]. However, no matter what the underlying mechanism of this finding is, antibodies binding within the 447–687 region of gB were non-neutralizing.

Antibodies which show potent *in vitro* neutralizing activity were found to bind to two previously unknown protein domains, namely AD-4 and AD-5. Because this was demonstrated in most donors, it is unlikely a sampling artifact. The antigenicity of these domains is also indicated by the fact that in randomly selected serum samples from HCMV-seropositive donors we found positivity rates of >90% for AD-4 and >50% for AD-5, which identified both domains as strongly antigenic structures on gB. Comparable positivity rates among sera from HCMV infected individuals have been reported for AD-1 and AD-2, respectively, and were confirmed in the current analysis [17]. A distinct difference between AD-4/AD-5 and AD-1/AD-2 is the functional antiviral activity of the domain specific antibodies. AD-1 is bound by virus neutralizing and non-neutralizing monoclonal antibodies which can compete for binding to the domain [35], [58]. Affinity purified AD-1-specific IgG fractions from pooled human sera were shown to have neutralizing capacity not exceeding 50%, indicating that during natural infection a considerable proportion of competing non-neutralizing antibodies are induced [36]. The incomplete neutralizing capacity of polyclonal anti-AD-1 antibodies has been suggested to provide the virus with an effective mechanism to evade the humoral immune response. Likewise, AD-2 harbors two different antibody binding sites which are bound by neutralizing and non-neutralizing antibodies, respectively [37]. The situation seems to be different for AD-4 and AD-5 in that almost all human monoclonal antibodies that have been obtained so far have potent virus neutralizing activity. Moreover, the affinity-purified AD-4-specific polyclonal human IgG fraction had a neutralizing titer that was comparable to the monoclonal antibodies with 50% neutralization in the low nanomolar range. Thus, it seems unlikely that AD-4 induces significant concentrations of non-neutralizing antibodies that compete with neutralizing antibodies for binding to the domain. Whether this correlation also holds true for AD-5 needs to be determined in further studies.

AD-4- and AD-5-specific mabs did not prevent virus attachment to fibroblasts indicating that neither type of antibody interferes with receptor binding of HCMV. However, they were capable of neutralizing infectious virus at a postadsorption step. In the case of HSV-1 it has been shown that neutralizing anti-gB murine mabs have different effector mechanisms. Antibodies that bind to the HSV-gB domain IV (corresponding to AD-1 in HCMV gB) block fusion of viral and cellular membranes but do not interfere with interaction of gB with gH/gL, the second constituent of the minimal herpesviral fusion complex. Murine mabs specific for the HSV-gB domains I and II (corresponding to AD-4 and AD-5 in HCMV gB) block interaction of gB with gH/gL and thereby inhibit fusion [59], [60]. Whether similar effector mechanisms apply for HCMV needs to be determined in further studies.

HCMV isolates from clinical samples exhibit extensive genetic variation and HCMV reinfections have been demonstrated to occur in seropositive individuals. In immunocompromised hosts, when the development of a *de novo* humoral immune response is impaired, reinfection with a different HCMV isolate might result in clinical symptoms, due to unrestricted replication of the ‘new’ virus strain [61]–[65]. However, we have no information on whether reinfection of seropositive individuals is inevitable upon contact with a different HCMV strain or whether some infected hosts develop an immune response which prevents reinfection. It will be important to analyze whether such “absolute controllers” exist and what the correlate of protection is. In the HIV field it has clearly been shown, that a small fraction of infected individuals can develop broadly neutralizing antibodies which control the viral mutants that develop during infection and prevent progression to disease [53], [66]. In the case of HCMV, it will be interesting to determine whether production of antibodies against the individual antigenic domains of gB or, for that matter additional envelope glycoproteins, correlates with reduced susceptibility to reinfection. Of note, the gB protein region harboring AD-4 and AD-5 is highly conserved between different HCMV isolates (Fig. S5) and is situated well outside the polymorphic protein segments that have been defined [67].

In summary, we have investigated the human antibody repertoire against gB using a recombinant protein that is currently used in vaccine trials. Our data reveal new antigenic sites on the protein, which are immunogenic during infection and, more importantly, target of potent antiviral antibodies. It will be interesting to compare our findings to the B-cell repertoire against gB as it is produced during infection since this may enable us to draw conclusions about the structural integrity of the gB vaccine antigen. Improving our understanding of the antigenic map of gB will be of value in the rational design of future vaccine antigens. Lastly, human mabs with potent and broadly neutralizing activity might be useful as biologicals in prophylaxis and/or therapy of HCMV infections.

## Materials and Methods

### Ethics statement

Ethics approval for the sample collection has been obtained from the Ethics Committee of the Medical Faculty of the Friedrich-Alexander Universität Erlangen-Nürnberg. Written informed consent was obtained from all donors.

### Cells and viruses

African green monkey kidney cells (Cos7) were cultured in Dulbecco’s modified Eagle’s medium (DMEM) (Invitrogen, Germany) supplemented with 10% fetal calf serum (FCS), glutamine (100 mg/l) and gentamicin (350 mg/l). HCMV was propagated in human fetal lung fibroblasts (MRC-5) or human foreskin fibroblasts (HFF) grown in DMEM supplemented with 10% FCS, glutamine and gentamicin as above. For immunofluorescence cells were grown on 13-mm glass coverslips in 24-well plates. HB15-UL84prluc represents a recombinant AD169-based HCMV which expresses the firefly luciferase gene under the control of the HCMV UL84 promoter.

### Flow cytometry analysis and cell sorting of glycoprotein B-specific memory B cells

Peripheral blood mononuclear cells (PBMCs) were isolated from peripheral blood of healthy, HCMV-positive volunteers using Ficoll-density gradient centrifugation. After B-cell enrichment using anti-human CD22-microbeads (Mitenyi Biotec, Bergisch Gladbach, Germany), B cells were labeled with the following reagents: 1. Anti-human CD19-PerCP (Dianova, Germany); 2. Anti-human CD27-PE (BD Bioscience Pharmigen, Switzerland); 3. anti-human IgG-FITC (Dianova, Germany); 4. Cy5-labeled glycoprotein B (Sanofi Pasteur, 100 ng per 1×10^6^ B cells). The gB protein was labeled with Cy5 using the FluoroLink-Ab Cy5 labelling kit (Amersham Pharmacia Biotech, Germany). gB-specific, IgG-positive memory B cells were either analyzed using FACSCalibur (Becton Dickinson, Germany) or isolated by sorting cells that fulfilled the criteria PerCP+/PE+/FITC+ and Cy5+. Cells were sorted at 5 cells/well, in 96 F-bottom microplates containing a confluent layer of irradiated feeder cells (HFF), using a MoFlo cell sorter (Cytomation, Germany). Sorted cells were grown in complete RPMI-1640 medium supplemented with 2 mM glutamine, 100 IU/ml penicillin, 100 µg/ml streptomycin, 50 µM 2-mercaptoethanol and 10% FCS (heat-inactivated) (PAN-Biotech, Germany) in the presence of EBV and CpG ODN 2006 as previously described [44]. After 3 weeks, the culture supernatants were screened for antigen recognition and/or virus neutralization. Supernatants were classified as neutralizing if at a 1:1 dilution the neutralization capacity exceeded 70% of input virus.

### Recombinant expression of IgG

To produce selected recombinant human monoclonal antibodies the Ig heavy and corresponding light chains were amplified by RT-PCR from clonally expanded activated memory B cells and cloned into eukaryotic expression vectors exactly as described by Tiller et al. [68]. The respective cloning vectors were kindly provided by H. Wardemann, Berlin. V gene usage and CDR sequences are supplied in Table S1.

### Virus neutralization assay

Fibroblasts (1×10^4^) in a volume of 100 µl were seeded in 96-well plates. HB15-UL84prluc (275 pfu) was preincubated with serial log_2_ dilutions of complement inactivated serum or mab in a volume of 50 µl in culture medium for 1 h at 37°C. The mixture was added to fibroblasts for 4 h. The inoculum was removed and the cells were incubated at 37°C for 48 h. Cells were lysed using 100 µl Glo Lysis Buffer (Promega, USA) per well. 30 µl of each cell lysate was placed in white 96-well LIA-plates. Per well, 50 µl assay buffer (15 mM KH_2_PO_4_, 25 mM glycylglycine, 1 M MgSO_4_, 0.5 M EGTA, 5 mM ATP, 1 mM DTT) was added. Luciferase activity was measured by injection of 50 µl D-luciferin (P.J.K., Germany) solution per well (in 25 mM glycylglycine, 1 M MgSO_4_, 0.5 M EGTA, 2 mM DTT and 0.05 mM D-luciferin) and detection of chemiluminescence was performed by an Orion Microplate Luminometer (Berthold Technologies, Germany). Neutralization assays using endothelial and epithelial cells were performed using the HCMV isolate TB40E as described [69]. The influence of complement on neutralization capacity of monoclonal antibodies was tested by inclusion of 5% rabbit complement (Cedarlane Labs, Canada) in the neutralization assay.

### Virus adsorption and penetration

Fibroblasts were seeded at 3×10^4^ cells per well in 96-well plates. HB15-UL84prluc was preincubated with individual mabs for 1 h at 37°C at concentrations ensuring complete neutralization. Cells and the virus/mab mixture were cooled to 4°C and the virus/mab mixture was added to the cells at a multiplicity of infection (m.o.i.) of 0.5. Following incubation for 1h at 4°C, cells were washed three times with ice-cold PBS and cell lysates were prepared by freezing/thawing. DNA was extracted from the lysates using a MagNA Pure LC (Roche, Germany) instrument and quantitative real-time PCR was performed on an ABI PRISM 7500. To control for recovery of cells, copy numbers of albumine DNA was determined in parallel to HCMV and HCMV copies were calculated per 1000 copies albumine. Primers : CMV 5′:GAGCAGACTCTCAGAGGATCGG; CMV 5′: AAGCGGCCTCTGATAACCAAG; Albumine 5′: GTGAACAGGCGACCATGCT; Albumine 3′: GCATGGAAGGTGAATGTTTCAG. For the penetration assay, precooled cells were preincubated with virus at a m.o.i. of 0.2 for 1 h at 4°C, washed twice with ice-cold PBS and incubated for 2 h at 37°C with log_10_ dilutions of mabs. After incubation, remaining mabs were removed by washing twice with PBS before cells were incubated for 48 h at 37°C. Subsequent steps were carried out according to the virus neutralization assay. Chemiluminescence of virus only was set to 100%.

### Plasmids

Construction of the expression plasmid coding for complete gB has been described previously [32]. Carboxyterminal truncated forms of gB were expressed using pcDNA3 as plasmid. Aminoterminal truncations were expressed using the vector pcUL132sigHA. This pcDNA3.1 based plasmid contains the coding sequence of the HCMV gpUL132 authentic signal sequence aa 1-27, followed by the coding sequence for the influenza hemagglutinin (HA) epitope YPYDVPDYA [70]. To express Dom II (AD-4), the nucleotide sequence coding for aa 112–132 and 344–438 was chemically synthesized by GeneArt, Germany. The two parts were joined by a nucleotide linker coding for the sequence Ile-Ala-Gly-Ser-Gly and inserted in pcUL132sigHA to give rise to pcAD-4. To express Dom I (pcAD-5) the nucleotide sequence coding for aa 133-343 were inserted into pcUL132sigHA. To generate plasmids for expression of AD-4-GST (Glutathion-S-transferase) fusion proteins in E. coli we used the expression vector pGEX-6P-1 (Pharmacia Biotech, Germany).

### Transient protein expression and image analysis

Cos7 cells grown on glass coverslips in 24-well plates were transfected with 0.8 µg of plasmid DNA using Lipofectamine (Invitrogen, Germany). 48 hours after transfection the cells were fixed and permeabilized with ice cold methanol. Primary antibodies were then added. Unbound primary antibody was removed by three washing steps using PBS. Binding of the primary antibody was detected with the appropriate secondary antibody conjugated with FITC (fluorescein isothiocyanate) (Dako, Germany). Counterstaining of cell nuclei was done with DAPI (4',6-diamidino-2-phenylindole). Images were collected using a Zeiss Axioplan 2 fluorescence microscope fitted with a Visitron Systems charge-coupled device camera (Puchheim, Germany). Images were processed using MetaView software and Adobe Photoshop. Antibodies: gB-specific human mab C23 (TI-23) [41], gN-specific murine mab 14-16A [71], gH-specific murine mab SA4 [72], murine anti-HA (Sigma Aldrich, Germany) and murine anti-GST (BIOZOL, Germany).

### Preparation of AD-4-GST fusion protein and immunoaffinity chromatography

Plasmid DNA was used to transform E. coli DH10B for expression of GST fusion proteins. The respective fusion proteins were induced and the soluble form of the protein was purified from E. coli lysates according to the manufacturer’s instructions. To prepare an affinity matrix, 2.6 mg of purified AD-4-GST fusion protein was dialysed against coupling buffer and conjugated to AminoLink Plus Coupling Resin (Thermo Fisher Scientific, USA) according to the manufacturer's instructions. Four ml of a HCMV hyperimmune globuline preparation, diluted 1∶3 (v/v) with PBS, was passed over 2 ml antigen-coupled beads, followed by extensive washing with PBS. Bound IgG was eluted with 0.2 M Glycin-HCl, pH 3.0, in 1 ml fractions and fractions were dialysed against PBS. Total IgG concentration was determined by an ELISA. In brief, polystyrene 96-well plates were coated with 100 ng AffiniPure goat anti-human IgG, Fcγ-specific (Jackson Immuno Research, USA) in 0.5 M carbonate buffer, pH 9.6, overnight at 4°C. Serial log_2_ dilutions of the eluted fractions in a volume of 50 µl were added and bound IgG was detected by using a polyclonal peroxidase-conjugated goat F(ab)_2_-fragment anti-human IgG, Fcγ-specific (Jackson Immuno Research, USA). A human IgG preparation (Jackson Immuno Research, USA) with known concentration was used as standard.

### ELISA

The following gB-specific antigens were used: gB, AD-1, containing aa 484–650 of gB, AD-2, containing aa 68–80 and AD-4-GST. Proteins were diluted between 25 ng and 200 ng (depending on antigen) in 0.5 M sodium carbonate buffer, pH 9.6, or in 6 M urea (AD-1) and 50 µl was used to coat microtiter plates overnight at 4°C. All subsequent steps were carried out at room temperature. Reaction wells were rinsed with PBS supplemented with 0.1% Tween 20 and blocked for 2 h with PBS containing 2% FCS. Plates were again rinsed with PBS supplemented with 0.1% Tween 20 and incubated with mabs, human serum or polyclonal eluted antibody fractions (50 µl/well) for 2 h. Unbound antibody was removed by washing and peroxidase-conjugated anti-human or anti-mouse IgG (Dako, Germany) was added at an appropriate dilution for 1 h. The plate was washed and 100 µl TMB (tetramethylbenzidine) peroxidase substrate, diluted 1∶1 in peroxidase substrate solution B (KPL, USA), was added for 5 min. The reaction was stopped by the addition of 100 µl 1 M H_3_PO_4_ and the OD_450_ was determined using Emax microplate reader (Eurofins MWG Operon, Germany). Dilution of all antibodies was done in PBS with 2% FCS. In all assays involving gB fusion proteins, the respective prokaryotic fusion partner was assayed in parallel and the optical density subtracted from values obtained with the gB fusion protein. AD-5-specific antibodies in human sera were measured in a capture ELISA. 96 well plates were coated with 125 ng/well of an anti-HA monoclonal antibody (Sigma, Germany) and blocked for 2 h with PBS containing 2% FCS. The plates were incubated with culture supernatant from cells that had been transfected with pcAD-5 six days before. Plates were rinsed and incubated with human sera in a 1∶50 dilution for 2 h at 37°C. The plates were washed and developed as described above.

### Generation of the HCMV gB model

The model of the HCMV gB structure was generated by standard homology modelling procedures using the program MODELLER [73], based on a sequence alignment with the template structure of HSV-1 gB [46]. Two loop regions (Val_306_ to Glu_317_ and Leu_439_ to His_468_ of HCMV gB) were not resolved in the reference structure and could therefore not be modelled. All images were generated with Accelrys DS Visualizer v2.0.1. The quality of the model was validated using ProSA [74] and PROCHECK [75]. ProSA analysis reveals that the overall model quality (Z-score  =  −6.22) is quite similar to that of the template crystal structure (Z-score  =  −7.84). Both values are within the range of *z*-scores typically found for crystal structures of proteins of similar size. In addition, the residue energy profiles of template and target structure are very similar indicating that the modelling did not place amino acids in an unfavourable environment. In addition, analysis of the backbone geometry shows that that 87.5% of all residues of HCMV gB are located in the most favourable regions of the Ramachandran Plot. This value corresponds to a crystal structure with 2.0–2.5 Å resolution. The HCMV gB model will be made available by the authors upon request.

### Accession numbers

GenBank accession numbers for the individual heavy and light chain nucleotide sequences of the recombinantly expressed IgG molecules are JF806449-JF806467.

## Supporting Information

Figure S1 **Staining of gB-specific, IgG-positive memory B cells for frequency analysis and sorting by flow cytometry.** (A) B cells were stained with fluorochrome-labeled antibodies against CD19, CD27, IgG and with Cy5-labeled gB. (B) gB-specificity as percentage of all IgG-bearing memory B cells among HCMV-positive and –negative blood donors. Horizontal bar: mean value; Mann-Whitney Test.
(TIF)

Figure S2 **Serologic characterization of donors and neutralizing capacity of B-cell supernatants.** (A) Reactivity of donor sera with gB and antigenic domains of gB in ELISA. The ELISA was carried out as described in Material and Methods. HCMV-negative sera (MW, CB, FK) are included as controls. (B) Neutralization capacity of sera selected for the repertoire analysis. The recombinant AD169-derived virus was used. The analysis was repeated twice with similar results. (C) Neutralization capacity of B-cell supernatants from five individual donors. B-cell supernatants were incubated with HCMV for 1 h before addition to fibroblasts. The percentage of neutralization is plotted as a function of the IgG concentration. Shown are all B-cell supernatants which exceeded 50% neutralization at the highest IgG concentration. Different colors indicate gB domain specificity as depicted in Fig. 3. Every antibody was tested at least two times.
(TIF)

Figure S3 **Characterization of the recombinant Dom II protein.** (A) PAGE analysis of the Dom II-GST fusion protein. The protein was expressed and purified as described in Material and Methods. (B) Kinetic data for binding of SM5-1 to gB and Dom II-GST. SM5-1 was used as Fab-fragment generated by papain digestion. Kinetic experiments were performed at 25°C using a Biacore T100 (GE Healthcare, Germany). gB and Dom II-GST proteins were captured on the Series S Sensor Chip CM5 (GE Healthcare, Germany) using *N*-hydroxysuccinimide-*N*-ethyl-*N-*dimethylamino-propyl-carboimide chemistry. PBS, 0.05% P20 was used as the running buffer. Approximately 300 RU of Dom II-GST and GST as reference surface were captured with a contact time of 400 sec and a flow rate of 10 µl/min. Different concentrations of SM5-1 (33.33, 11.11 and 3.7 nM) were injected with a contact time of 90 sec, dissociation time of 600 sec and a flow rate of 30 µl/min. The sensor surface was regenerated between each binding reaction with 10 mM glycine (pH 2.0) with a contact time of 20 sec and a flow rate of 30 µl/min. The kinetics were fitted to a 1∶1 binding model. *k_a_*, apparent association rate constant; *k_d_*, apparent dissociation rate constant; *K_D_*, apparent dissociation equilibrium. (C) Circular dichroism (CD) measurement of Dom II protein demonstrating structural folding. PreScission Protease (GE Healthcare, Germany) was used to cleave the GST-Tag. Dom II protein at a concentration of 7.8 µM was dialyzed against 20 mM sodium phosphate buffer (pH 7.0) and filtrated. CD measurement was performed at 20°C using a Jasco J-815 CD Spectrometer (Jasco, Japan) and a cuvette with 0.1 cm path length. Spectrum was registered from 185 to 260 nm and was corrected for the contribution of phosphate buffer. Spectrum was accumulated eight times with a band width of 1.0 nm and a sensitivity of 100 mdeg. The scan speed was 20 nm/min, the time response 1 sec and the data pitch 0.1 nm.
(TIF)

Figure S4 **Quality controls of Dom II-specific polyclonal antibody preparation.** (A) The affinity purified polyclonal antibody fraction is specific for Dom II of gB. ELISA plates were coated with gB, AD-1, AD-2 and Dom II, respectively, and tested with various antibodies. Dom II poly: affinity purified IgG fraction, Serum pool pre: Serum pool before affinity purification, Serum post: Serum pool after affinity purification, AD-2-specific mab: C23, anti-AD-1-specific mab: 89-104, anti-GST: murine mab specific for GST. (B) The affinity purified polyclonal antibody fraction does not contain detectable antibodies against additional envelope glycoproteins of HCMV. Cos7 cells were transfected with the plasmids indicated in the top row. 48 h later the cells were fixed and incubated with the affinity purified IgG fraction (upper panel) and control antibodies (lower panel). Binding of the primary antibody was detected by incubation with appropriate FITC-conjugated secondary antibody. Anti-HA: mouse mab specific for HA, C23: anti-AD-2 human mab, 14-16A: mouse mab specific for the gM/gN complex, SA4: mouse mab specific for gH. Antibody purification was performed twice with similar results.
(TIF)

Figure S5 **Sequence alignment of HCMV strains and clinical isolates.** Full length HCMV protein sequences from Genbank and EMBL databases (accession numbers on the left) were aligned to the HCMV TB40 strain. The regions for AD-2, domain I, domain II as well as AD-1 are depicted. Two regions of hyper-variability lie close to the N-terminus and C-terminal from domain II in a linker region. The protein alignment was performed with the Geneious software v4.8 (Drummond AJ, Ashton B, Buxton S, Cheung M, Heled J, Kearse M, Moir R, Stones-Havas S, Thierer T, Wilson A (2010) from http://www.geneious.com.
(TIF)

Table S1 **V gene usage and CDR sequences of monoclonal anti-gB antibodies.** The amino acid sequences of the CDR regions of heavy and light chain genes of monoclonal anti-gB antibodies are shown. * Assignments of V-genes and CDR regions were performed with IMGT/V-QUEST (http://www.imgt.org). The light chain sequence of clone SM11-17 could not be obtained.
(PDF)
