# Supplementary figures and images for: B Cell Repertoire Analysis Identifies New Antigenic Domains on Glycoprotein B of Human Cytomegalovirus which Are Target of Neutralizing Antibodies
Source: PLoS Pathog. 2011 Aug 11;7(8):e1002172. doi: 10.1371/journal.ppat.1002172 (PMC3154849; doi:10.1371/journal.ppat.1002172)

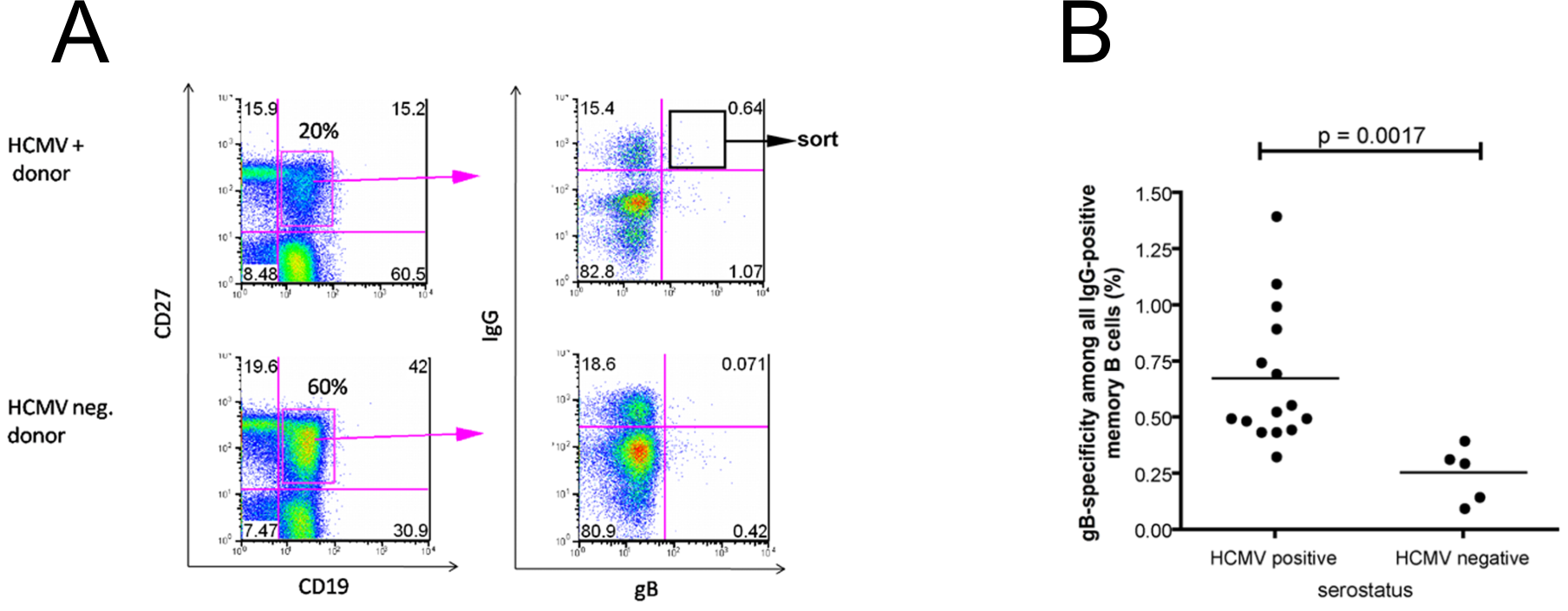

Supplement: Figure S1 — Staining of gB-specific, IgG-positive memory B cells for frequency analysis and sorting by flow cytometry. (A) B cells were stained with fluorochrome-labeled antibodies against CD19, CD27, IgG and with Cy5-labeled gB. (B) gB-specificity as percentage of all IgG-bearing memory B cells among HCMV-positive and –negative blood donors. Horizontal bar: mean value; Mann-Whitney Test. (TIF) [file ppat.1002172.s001.tif]

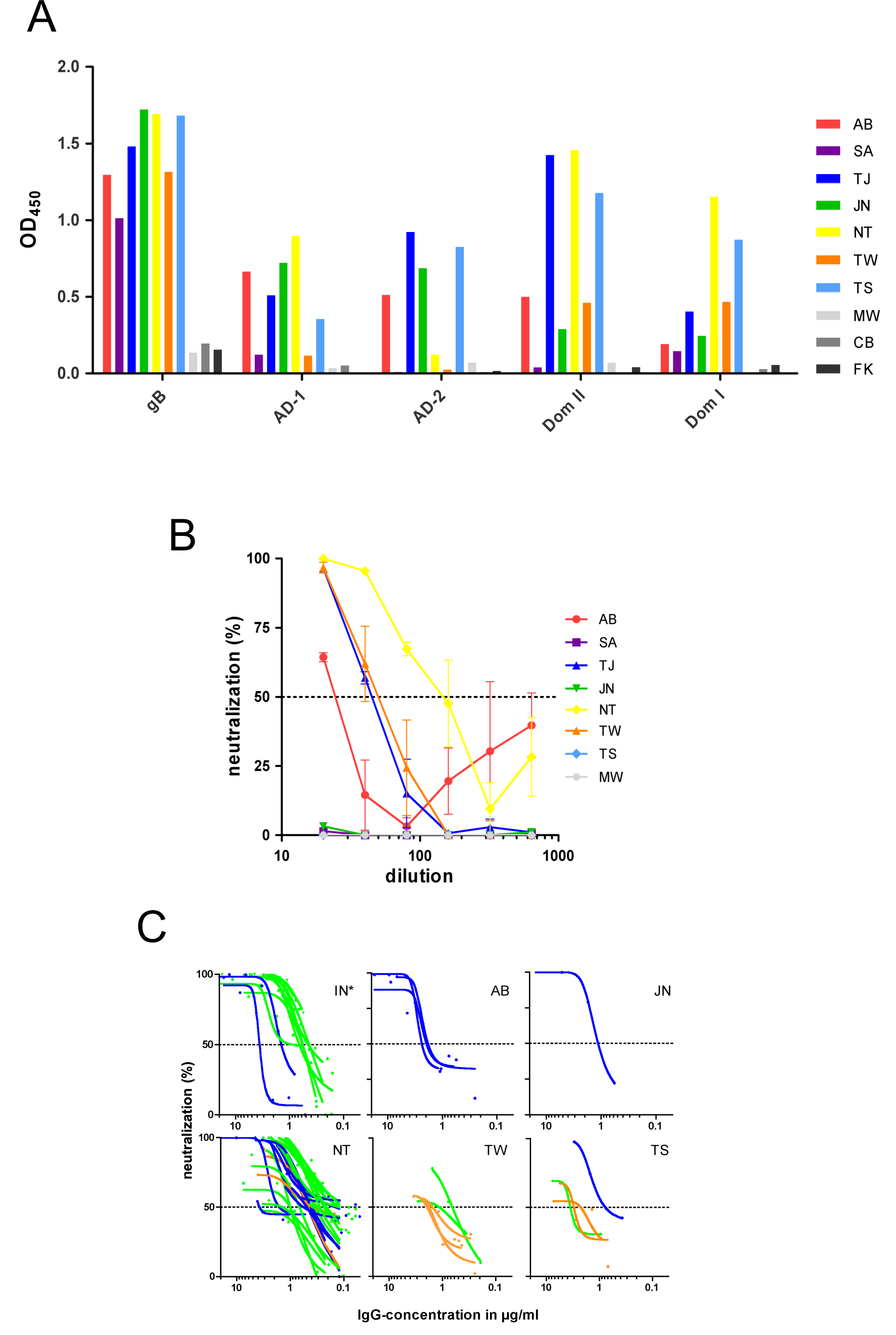

Supplement: Figure S2 — Serologic characterization of donors and neutralizing capacity of B-cell supernatants. (A) Reactivity of donor sera with gB and antigenic domains of gB in ELISA. The ELISA was carried out as described in Material and Methods. HCMV-negative sera (MW, CB, FK) are included as controls. (B) Neutralization capacity of sera selected for the repertoire analysis. The recombinant AD169-derived virus was used. The analysis was repeated twice with similar results. (C) Neutralization capacity of B-cell supernatants from five individual donors. B-cell supernatants were incubated with HCMV for 1 h before addition to fibroblasts. The percentage of neutralization is plotted as a function of the IgG concentration. Shown are all B-cell supernatants which exceeded 50% neutralization at the highest IgG concentration. Different colors indicate gB domain specificity as depicted in Fig. 3. Every antibody was tested at least two times. (TIF) [file ppat.1002172.s002.tif]

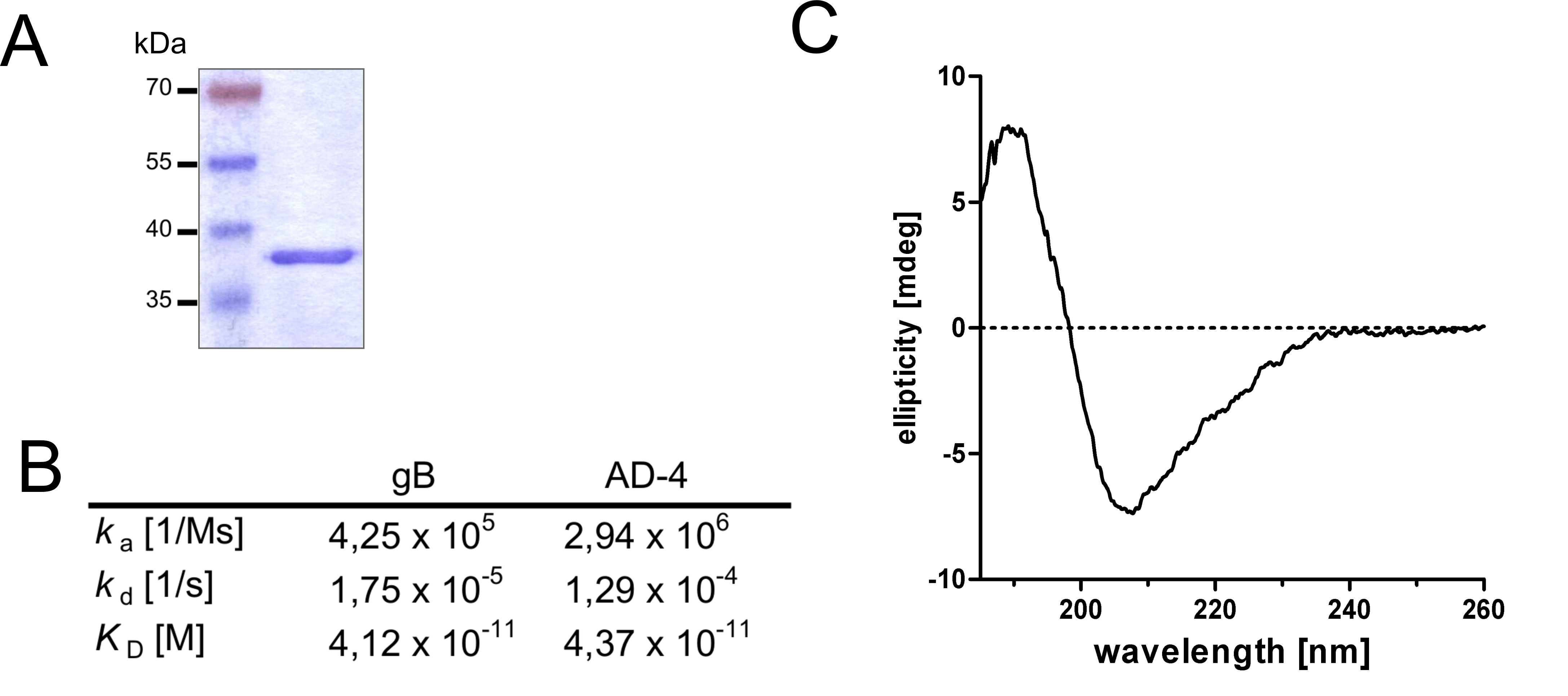

Supplement: Figure S3 — Characterization of the recombinant Dom II protein. (A) PAGE analysis of the Dom II-GST fusion protein. The protein was expressed and purified as described in Material and Methods. (B) Kinetic data for binding of SM5-1 to gB and Dom II-GST. SM5-1 was used as Fab-fragment generated by papain digestion. Kinetic experiments were performed at 25°C using a Biacore T100 (GE Healthcare, Germany). gB and Dom II-GST proteins were captured on the Series S Sensor Chip CM5 (GE Healthcare, Germany) using N-hydroxysuccinimide-N-ethyl-N-dimethylamino-propyl-carboimide chemistry. PBS, 0.05% P20 was used as the running buffer. Approximately 300 RU of Dom II-GST and GST as reference surface were captured with a contact time of 400 sec and a flow rate of 10 µl/min. Different concentrations of SM5-1 (33.33, 11.11 and 3.7 nM) were injected with a contact time of 90 sec, dissociation time of 600 sec and a flow rate of 30 µl/min. The sensor surface was regenerated between each binding reaction with 10 mM glycine (pH 2.0) with a contact time of 20 sec and a flow rate of 30 µl/min. The kinetics were fitted to a 1∶1 binding model. ka, apparent association rate constant; kd, apparent dissociation rate constant; KD, apparent dissociation equilibrium. (C) Circular dichroism (CD) measurement of Dom II protein demonstrating structural folding. PreScission Protease (GE Healthcare, Germany) was used to cleave the GST-Tag. Dom II protein at a concentration of 7.8 µM was dialyzed against 20 mM sodium phosphate buffer (pH 7.0) and filtrated. CD measurement was performed at 20°C using a Jasco J-815 CD Spectrometer (Jasco, Japan) and a cuvette with 0.1 cm path length. Spectrum was registered from 185 to 260 nm and was corrected for the contribution of phosphate buffer. Spectrum was accumulated eight times with a band width of 1.0 nm and a sensitivity of 100 mdeg. The scan speed was 20 nm/min, the time response 1 sec and the data pitch 0.1 nm. (TIF) [file ppat.1002172.s003.tif]

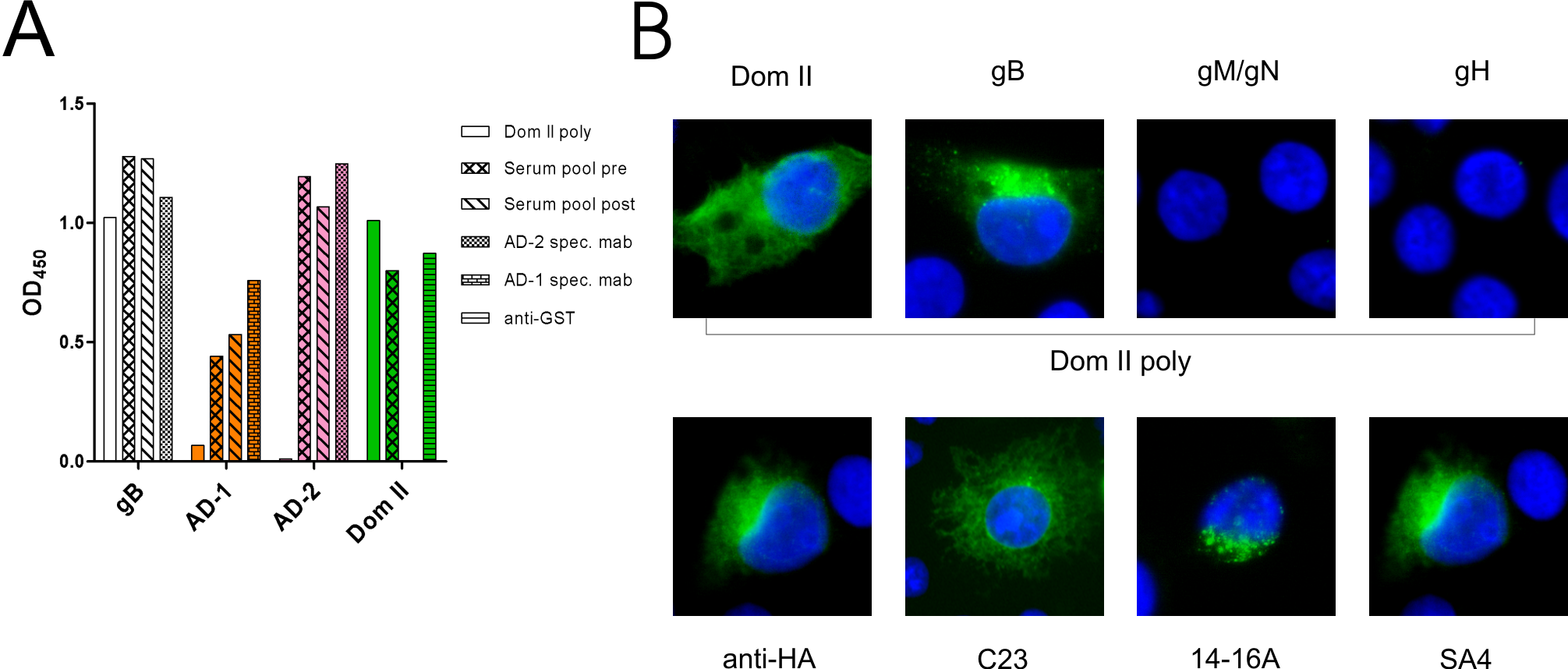

Supplement: Figure S4 — Quality controls of Dom II-specific polyclonal antibody preparation. (A) The affinity purified polyclonal antibody fraction is specific for Dom II of gB. ELISA plates were coated with gB, AD-1, AD-2 and Dom II, respectively, and tested with various antibodies. Dom II poly: affinity purified IgG fraction, Serum pool pre: Serum pool before affinity purification, Serum post: Serum pool after affinity purification, AD-2-specific mab: C23, anti-AD-1-specific mab: 89-104, anti-GST: murine mab specific for GST. (B) The affinity purified polyclonal antibody fraction does not contain detectable antibodies against additional envelope glycoproteins of HCMV. Cos7 cells were transfected with the plasmids indicated in the top row. 48 h later the cells were fixed and incubated with the affinity purified IgG fraction (upper panel) and control antibodies (lower panel). Binding of the primary antibody was detected by incubation with appropriate FITC-conjugated secondary antibody. Anti-HA: mouse mab specific for HA, C23: anti-AD-2 human mab, 14-16A: mouse mab specific for the gM/gN complex, SA4: mouse mab specific for gH. Antibody purification was performed twice with similar results. (TIF) [file ppat.1002172.s004.tif]

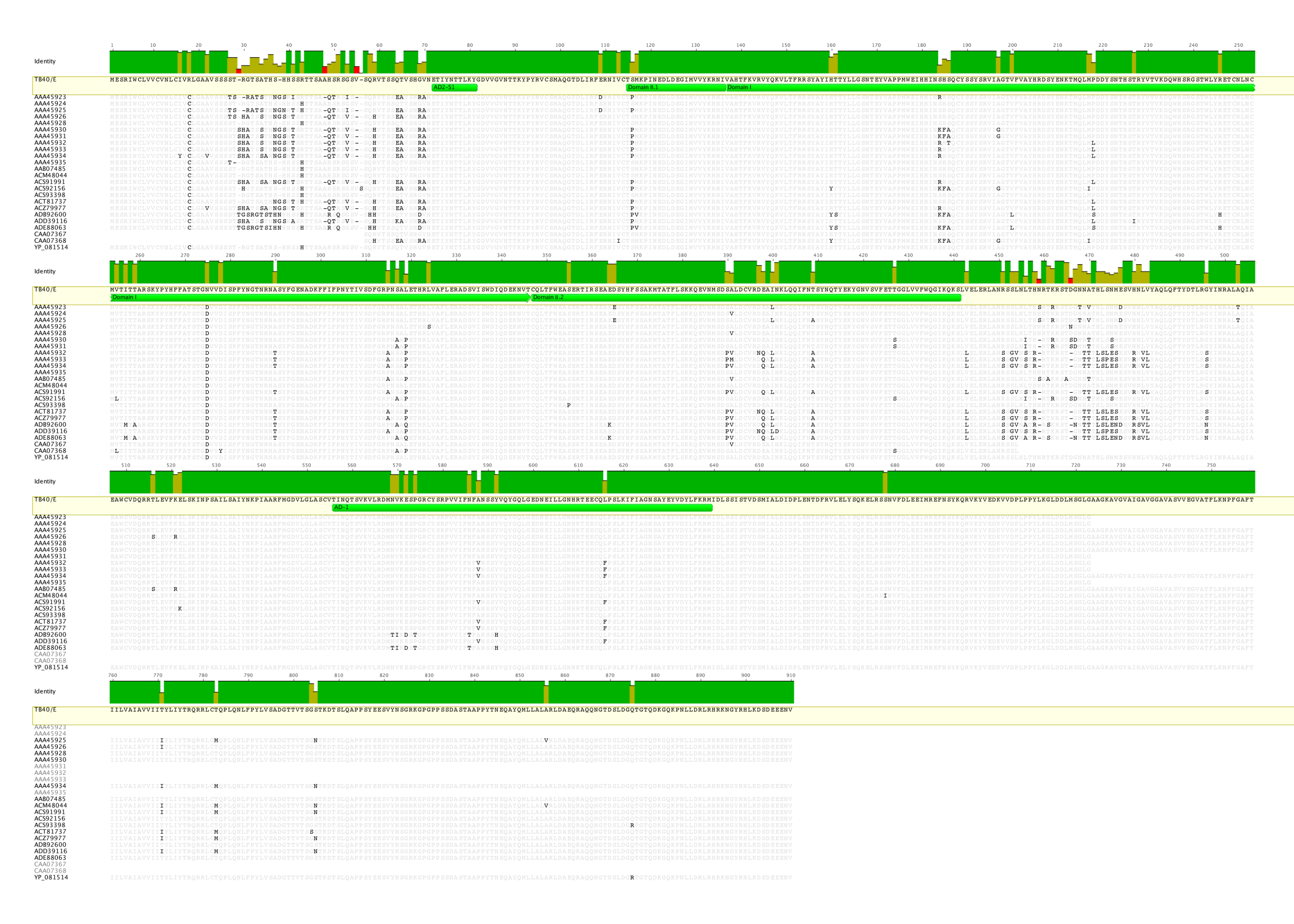

Supplement: Figure S5 — Sequence alignment of HCMV strains and clinical isolates. Full length HCMV protein sequences from Genbank and EMBL databases (accession numbers on the left) were aligned to the HCMV TB40 strain. The regions for AD-2, domain I, domain II as well as AD-1 are depicted. Two regions of hyper-variability lie close to the N-terminus and C-terminal from domain II in a linker region. The protein alignment was performed with the Geneious software v4.8 (Drummond AJ, Ashton B, Buxton S, Cheung M, Heled J, Kearse M, Moir R, Stones-Havas S, Thierer T, Wilson A (2010) from http://www.geneious.com. (TIF) [file ppat.1002172.s005.tif]
