# Supplementary material for: B Cell Repertoire Analysis Identifies New Antigenic Domains on Glycoprotein B of Human Cytomegalovirus which Are Target of Neutralizing Antibodies
Source: PLoS Pathog. 2011 Aug 11;7(8):e1002172. doi: 10.1371/journal.ppat.1002172 (PMC3154849; doi:10.1371/journal.ppat.1002172)
Supplement: Table S1 — V gene usage and CDR sequences of monoclonal anti-gB antibodies. The amino acid sequences of the CDR regions of heavy and light chain genes of monoclonal anti-gB antibodies are shown. * Assignments of V-genes and CDR regions were performed with IMGT/V-QUEST (http://www.imgt.org). The light chain sequence of clone SM11-17 could not be obtained. (PDF) [file ppat.1002172.s006.pdf]

Supplementary Table 1: V gene usage and CDR sequences of monoclonal anti-gB antibodies

| Clone | V-GENE and allele* | CDR1* | CDR2* | CDR3* |
|---|---|---|---|---|
| heavy chain | | | | |
| SM10 | IGHV4-39*01 | GGSIGVGDSY | IYFTGTT | ARHAYDFWVRGVSWIAP |
| SM12 | IGHV4-59*01 | GGSMSSYY | IYYSGST | ARDVRSSSPPVYYYYGMDV |
| 2C2 | IGHV4-59*01 | GAPIRSYY | INTNGRS | ATASQHRYDSLTGSYRYYPYVMDV |
| 1G2 | IGHV4-39*01 | GASIDRSTYY | IYYNGRA | ATRWNYFFDFDY |
| SM1-6 | IGHV1-2*02 | GYTFTGYY | INPNSGGT | ARDGAKTVTTSGMSLLYYYDVMDI |
| SM3-1 | IGHV1-2*02 | GYTFTDYY | INPHSGGT | ARDGAKTVTTFGASLLYYYDIMDV |
| SM4-5 | IGHV1-2*02 | GFTLSDHY | INPQSGGT | ARDGAKTVSNSGLGLLYYHSRLDA |
| SM5-1 | IGHV1-2*02 | GYSLKDHY | INPQSGGT | ARDGAKTVSNSGLSLLYYHNRLDA |
| SM6-5 | IGHV1-2*02 | GYTFTDYY | INPNTGGT | ARDGAKTMTTSGLSLLYYYDVMDV |
| SM11-17 | IGHV1-2*02 | GYTFTDFY | INPHSGGT | ARDGAKTVTTSGMSLLYYYDVMDI |
| light chain | | | | |
| SM10 | IGKV2-28*01 | QTLLHSNGNNY | YAS | MQALQSPLT |
| SM12 | IGKV2-28*01 | QSLLHSNGYNY | LGS | MQALQTPRT |
| 2C2 | IGKV1-33*01 | QDIRKS | DAS | QHYDNFPPT |
| 1G2 | IGLV1-47*01 | SSNIETNY | RNN | GTWDDNSWV |
| SM1-6 | IGLV1-51*01 | SSNIGKNY | DNN | GTWDSSLSVV |
| SM3-1 | IGLV1-51*01 | SSNIGKNY | DNN | ATWDRSLSVV |
| SM4-5 | IGLV1-51*01 | RSNIGKNY | DNN | GTWDSDLSAYV |
| SM5-1 | IGLV1-51*01 | SSNIGKNY | DNN | GTPDRSLSVI |
| SM6-5 | IGLV1-51*01 | SSNIGKNY | DNN | ATWDSSLGVV |

* Assignments of V-genes and CDR regions were performed with IMGT/V-QUEST (http://www.imgt.org).
The light chain sequence of clone SM11-17 could not be obtained.
